# BioTM Buzz (Volume 4, Issue 1)

**DOI:** 10.1002/btm2.10125

**Published:** 2018-12-30

**Authors:** Aaron C. Anselmo

**Affiliations:** ^1^ Division of Pharmacoengineering and Molecular Pharmaceutics, Eshelman School of Pharmacy University of North Carolina at Chapel Hill Chapel Hill NC

## SPECIAL ISSUE: TRIBUTE TO ROBERT LANGER AND NICHOLAS PEPPAS

In this Special Issue of *Bioengineering & Translational Medicine*, we honor two luminaries in the AIChE community who pioneered the field of drug delivery and biomaterials, Professors Robert Langer, and Nicholas Peppas. These two giants, born within 4 days of each other in two different continents, met during their graduate school at the Massachusetts Institute of Technology, and have since been collectively championing the field. Professor Peppas is currently the Cockrell Family Regents Chaired Professorship in Engineering, Medicine and Pharmacy at the University of Texas, Austin and Professor Langer is currently the David H. Koch Institute Professor at the Massachusetts Institute of Technology.

Thanks to the pioneering efforts of these two individuals, drug delivery and biomaterials are now integral elements of Chemical Engineering research and education. The early contributions from these two pioneers established the very foundation of the field. Professor Langer established for the first time that proteins could be encapsulated in polymeric matrices and released in a sustained manner.^1^ Professor Peppas published several seminal papers establishing the quantitative principles for describing drug release from porous matrices and hydrogels.^2^ They both went on to make numerous groundbreaking contributions which have introduced new concepts in bioengineering, novel materials for biomedical applications, and new fundamental insights into biological systems. Together, their efforts have shaped the scholastic, academic, and technological landscape of drug delivery and biomaterials as we know today. Their pioneering contributions have also paved the way for the societies and journals that have further advanced the field. Individually, their impact on the field, as measured by any quantifiable means such as publications, citations, or patents is truly remarkable. However, when one looks at it collectively, the numbers are simply mind‐boggling and indicative of a truly remarkable time in the history of the field.

AIChE and SBE have been deeply appreciative and grateful for the contributions of Professors Peppas and Langer. Over the years, AIChE has celebrated Professors Peppas and Langer with its most prestigious awards that include the Founders Award for Outstanding Contributions to Chemical Engineering, William H. Walker Award, Institute Lecturer, Food, Pharmaceuticals and Bioengineering Award, and the Materials Engineering and Sciences Award from AIChE as well as the Jay Bailey Award from SBE. Most notably, both were listed among the “One Hundred Chemical Engineers of the Modern Era by AIChE” on the occasion of the AIChE centennial.

While the scholarly impact of Professors Langer and Peppas is transformative, we make a particular note of their dedication to mentoring students and post‐docs. Over the past 42 years, Professors Langer and Peppas have tirelessly recruited and mentored a large number of trainees who are advancing various frontiers of bioengineering and biotechnology in their own ways in academia as well as industry. Fittingly, this special issue features articles from some of their past students, as well as their colleagues and admirers.

Professor Peppas' and Langer's pioneering research, along with their active and inspiring leadership has advanced the visibility of drug delivery and biomaterials in the scientific landscape, especially within Chemical Engineering, and has inspired young professionals to follow their paths. The work featured in this issue provides a snapshot of the exciting work that has been inspired by them.

## MAINTAINING STEM CELLS PROPERTIES AFTER SERIAL EXPANSION

Human mesenchymal stem/stromal cells (hMSCs) have the potential to differentiate into various cell types that include bone, fat, muscle, and epithelial cells. hMSCs can also secrete cytokines and chemokines that control immune responses and thus promote wound healing. As such, hMSC are studied in clinical trials for treatment of graft versus host disease, myocardial infarctions, and tissue regeneration; this highlights the utility and potential of hMSCs in improving clinical care for a number of diseases/disorders. A significant challenge that has limited hMSC use beyond clinical trials are the issues related to changes in therapeutic function of hMSCs following ex vivo expansion. Since human studies require upward of millions of cells per kg, approaches to understand and control hMSC function during ex vivo expansion are needed. In this issue of *Bioengineering & Translational Medicine*, a team of engineers led by Professor Kristi Anseth in the Department of Chemical and Biological Engineering at the University of Colorado, Boulder, report a novel approach to rescue hMSC regenerative properties after culture and passaging on standard polystyrene substrates. Specifically, hMSCs proliferation rates, mechanosensing ability, cell surface marker expression, and secretory profiles that were diminished following culture on polystyrene could be recovered when subsequently cultured on soft poly(ethylene glycol) hydrogels.

DOI: 10.1002/btm2.10104

## DECELLULARIZED EXTRACELLULAR MATRICES FOR BONE AND CARTILAGE ENGINEERING

Recreating the complex tissue environment that exists in bone or cartilage has limited regenerative therapies for bone/cartilage injuries. Although synthetic materials can recreate key aspects of these tissues, there remains a significant challenge in sufficiently mimicking their complexities. Researchers from the lab of Professor Antonios Mikos in the Department of Bioengineering at Rice University, review how decellularization of the extracellular matrix in bone and cartilage can be used to form scaffolds, particles, and facilitate the secretion of supplementary factors for improved bone and cartilage tissue engineering. This review highlights approaches to achieve decellularization, postdecellularization processing methods, applications of decellularized extracellular matrices, and concludes with limitations and future areas of interest for the use of decellularized extracellular matrices in tissue engineering.

DOI: 10.1002/btm2.10110
